# Expression Quantitative Trait Loci Are Highly Sensitive to Cellular Differentiation State

**DOI:** 10.1371/journal.pgen.1000692

**Published:** 2009-10-16

**Authors:** Alice Gerrits, Yang Li, Bruno M. Tesson, Leonid V. Bystrykh, Ellen Weersing, Albertina Ausema, Bert Dontje, Xusheng Wang, Rainer Breitling, Ritsert C. Jansen, Gerald de Haan

**Affiliations:** 1 Department of Cell Biology, Section Stem Cell Biology, University Medical Center Groningen, University of Groningen, Groningen, The Netherlands; 2 Groningen Bioinformatics Centre, Groningen Biomolecular Sciences and Biotechnology Institute, University of Groningen, Haren, The Netherlands; 3 Department of Anatomy and Neurobiology, University of Tennessee Health Science Center, Memphis, Tennessee, United States of America; 4 Institute of Bioinformatics, Zhejiang University, Hangzhou, China; Georgia Institute of Technology, United States of America

## Abstract

Genetical genomics is a strategy for mapping gene expression variation to expression quantitative trait loci (eQTLs). We performed a genetical genomics experiment in four functionally distinct but developmentally closely related hematopoietic cell populations isolated from the BXD panel of recombinant inbred mouse strains. This analysis allowed us to analyze eQTL robustness/sensitivity across different cellular differentiation states. Although we identified a large number (365) of “*static*” eQTLs that were consistently active in all four cell types, we found a much larger number (1,283) of “*dynamic*” eQTLs showing cell-type–dependence. Of these, 140, 45, 531, and 295 were preferentially active in stem, progenitor, erythroid, and myeloid cells, respectively. A detailed investigation of those *dynamic* eQTLs showed that in many cases the eQTL specificity was associated with expression changes in the target gene. We found no evidence for target genes that were regulated by distinct eQTLs in different cell types, suggesting that large-scale changes within functional regulatory networks are uncommon. Our results demonstrate that heritable differences in gene expression are highly sensitive to the developmental stage of the cell population under study. Therefore, future genetical genomics studies should aim at studying multiple well-defined and highly purified cell types in order to construct as comprehensive a picture of the changing functional regulatory relationships as possible.

## Author Summary

Blood cell development from multipotent hematopoietic stem cells to specialized blood cells is accompanied by drastic changes in gene expression for which the triggers remain mostly unknown. Genetical genomics is an approach linking natural genetic variation to gene expression variation, thereby allowing the identification of genomic loci containing gene expression modulators (eQTLs). In this paper, we used a genetical genomics approach to analyze gene expression across four developmentally close blood cell types collected from a large number of genetically different but related mouse strains. We found that, while a significant number of eQTLs (365) had a consistent “*static*” regulatory effect on gene expression, an even larger number were found to be very sensitive to cell stage. As many as 1,283 eQTLs exhibited a “*dynamic*” behavior across cell types. By looking more closely at these *dynamic* eQTLs, we show that the sensitivity of eQTLs to cell stage is largely associated with gene expression changes in target genes. These results stress the importance of studying gene expression variation in well-defined cell populations. Only such studies will be able to reveal the important differences in gene regulation between different cell types.

## Introduction

Genetical genomics uses quantitative genetics on a panel of densely genotyped individuals to map genomic loci that modulate gene expression [1]. The quantitative trait loci identified in this manner are referred to as expression quantitative trait loci, or eQTLs [2]. Most genetical genomics studies that have thus far been reported have analyzed single cell types or compared developmentally unrelated and distant cell types [3]–[8]. Here, we report the first application of genetical genomics to study eQTL dynamics across closely related cell types during cellular development. We show results that discriminate between eQTLs that are consistently active or “*static*” and those that are cell-type–dependent or “*dynamic*.”

We used the hematopoietic system as a model to analyze how the genome of a single stem cell is able to generate a large variety of morphologically and functionally distinct differentiated cells. Differentiation of hematopoietic stem cells towards mature, lineage-committed blood cells is associated with profound changes in gene expression patterns. The search for differentially expressed genes, most notably for those transcripts exclusively present in stem cells and not in their more differentiated offspring, has been successful and has provided valuable insight into the molecular nature of stem cell self-renewal [9]–[12]. Yet, complementary approaches were needed to elucidate the dynamic regulatory pathways that are underlying the robust differentiation program leading to blood cell production.

We describe a genetic analysis of variation in gene expression across four functionally distinct, but developmentally related hematopoietic cell populations. Our data reveal complex cell-stage specific patterns of heritable variation in transcript abundance, demonstrating the plasticity of gene regulation during hematopoietic cell differentiation.

## Results

### Genetic Regulation of Gene Expression

**Figure 1:**
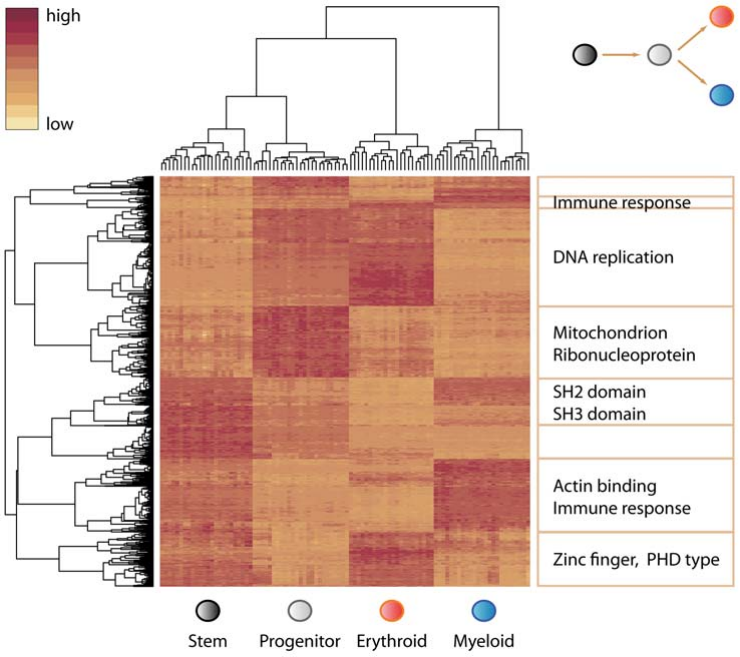
Mean expression levels for all probes in the four cell types. Unsupervised clustering including all probes for the 96 RNA samples follows cell type (top hierarchical tree), while clustering of the 876 most varying probes reveals distinct categories of genes that show cell-type–specific expression (left hierarchical tree). The heat map shows the expression patterns of those probes and selected enriched gene categories in each major cluster. Discriminatory genes are enriched in various functional classes, including SH2/SH3 domain containing transcription factors for stem cells, mitochondrial genes for progenitor cells, genes involved in DNA replication and zinc fingers for erythroid cells, and immunoglobulin type genes for myeloid cells (all *p*-values<0.05). For genes that belong to each of these clusters, see Table S1.

We evaluated genome-wide RNA transcript expression levels in purified Lin^−^Sca-1^+^c-Kit^+^ multi-lineage cells, committed Lin^−^Sca-1^−^c-Kit^+^ progenitor cells, erythroid TER-119^+^ cells, and myeloid Gr-1^+^ cells, isolated from the bone marrow of ∼25 genetically related and fully genotyped BXD – C57BL/6 (B6) X DBA/2 (D2) – recombinant inbred mouse strains [13]. In this study, we exploit the fact that the purified cell populations are closely related, sometimes just a few cell divisions apart on the hematopoietic trajectory. The Lin^−^Sca-1^+^c-Kit^+^ cell population contains all stem cells with long-term repopulating ability, but also includes multipotent progenitors that still have lymphoid potential. Although long-term repopulating stem cells are known to only make up a fraction of the Lin^−^Sca-1^+^c-Kit^+^ population, for simplicity we will refer to this population as stem cells. The Lin^−^Sca-1^−^c-Kit^+^ cell population does not contain stem cells and lymphoid precursors, but does include common progenitors of the myeloid and erythroid lineages [14]. Finally, TER-119^+^ cells and Gr-1^+^ cells are fully committed to the erythroid and myeloid lineages, respectively. Unsupervised clustering of the most varying transcripts demonstrated that each of the four cell populations could easily be recognized based on expression patterns across all four cell types (Figure 1 and Table S1).

We observed strong and biologically significant variation in gene expression during hematopoietic differentiation, independent of mouse strain. However, the genetical genomics strategy, in which we focus on *inter*-strain gene expression differences, allows for a far more comprehensive understanding of the genetic regulatory links underlying this variation. QTL mapping of gene expression traits allows us to identify eQTLs; genomic regions that have a regulatory effect on those expression traits. Two types of eQTLs can be distinguished, i.e., those that map near (less than 10 Mb from) the gene which encodes the transcript (*local*) and those that map elsewhere in the genome (*distant*) [15]. Together, *local* and *distant* eQTLs constitute a genome-wide overview of the gene regulatory networks that are active in the cell type under study. The strongest eQTLs were found for genes that were expressed only in mouse strains carrying one specific parental allele, suggesting that local regulatory elements are distinct between the two alleles. Cases of such allele-specific expression included *H2-Ob* and *Apobec3*. These transcripts were only detectable in strains that carried the B6 allele of the gene (see Figure S1A, S1B). A global view of heritable variation in gene expression indicated that the strongest eQTLs are not associated with the most highly expressed genes, and that for most probes the expression difference between the B6 and D2 alleles is small (see Figure S1C, S1D).

Since the focus of this project is to study the influence of cellular differentiation state on regulatory links, we used ANOVA to distinguish between “*static*” eQTLs that show consistent genetic effects across the four cell types and “*dynamic*” eQTLs that are sensitive to cellular state (i.e., eQTLs that have a statistically significant genotype-by-cell-type interaction). We further partitioned *dynamic* eQTLs into different categories on the basis of their dynamics along the differentiation trajectory.

### Cell-Type–Independent *Static* eQTLs

**Figure 2:**
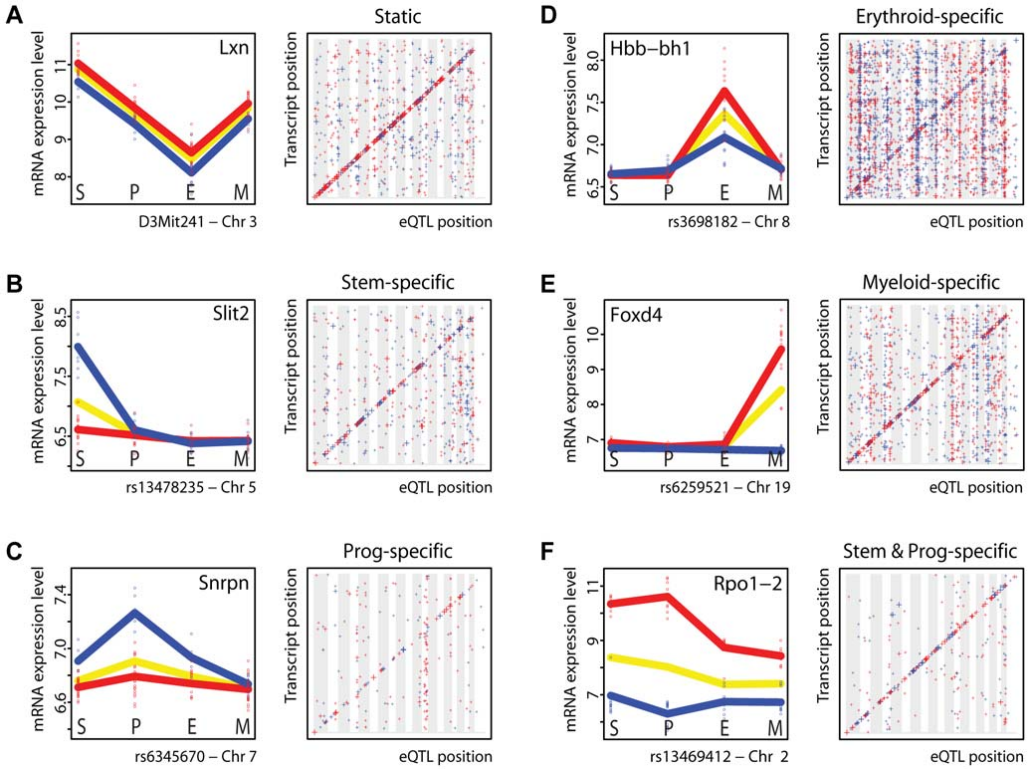
Identification of *static* and *dynamic* eQTLs. (A) Genome-wide identification of cell-type–independent *static* eQTLs. (Left panel) *Lxn* mRNA levels were analyzed in all 4 cell types. Each circle represents an individual sample (strain). The yellow line shows mean expression levels across all strains. The red and blue lines indicate mean *Lxn* expression levels in strains that carry the B6 or D2 *Lxn* allele, respectively. The genetic effect of parental alleles on *Lxn* expression levels was consistent in all cell types. (Right panel) Individual probes that detected a transcript that was consistently controlled by the same eQTL in all 4 cell types. The y-axis indicates the physical position of the encoding gene; the x-axis provides the genomic position of the marker with strongest linkage statistics. Vertical gray and white bandings indicate different chromosomes, ranging from chromosome 1 to X. The size of each symbol reflects the strength of the genetic association: eQTLs with *p*-values<10^−8^ are represented by the largest crosses; *p*-values between 10^−6^ and 10^−8^ are shown with medium crosses, while small crosses refer to eQTLs with *p*-values between 10^−4^ and 10^−6^. The color coding (red and blue) indicates the parental allele of the eQTL that caused a higher gene expression (B6 is red and D2 is blue). (B–E) Genome-wide identification of transcripts that are controlled by cell-type–specific eQTLs. (Left panels) Expression data for some transcripts that were affected by cell-type–specific eQTLs [(B) *Slit2* in stem cells, (C) *Snrpn* in progenitor cells, (D) *Hbb-bh1* in erythroid cells, and (E) *Foxd4* in myeloid cells]. (Right panels) Genome-wide distribution of eQTLs that were preferentially/uniquely detected in each of the four cell populations. (F) Transcripts that were controlled by eQTLs in both stem and progenitor cells. An example is *Rpo1-2*. Full lists of all genes belonging to the eQTL (sub)categories shown here are provided in Table S2.

The first eQTL category comprises genes that have *static* eQTLs across all four cell types under study. Variation in *Lxn* expression is shown as a representative example (Figure 2A, left panel). *Lxn* expression has previously been shown to be higher in B6 stem cells compared to D2 stem cells, and to be negatively correlated with stem cell numbers [16]. In our dataset *Lxn* showed clear expression dynamics (it was most highly expressed in stem cells), and was indeed more strongly expressed in cells carrying the B6 allele, but the expression difference between mice carrying the B6 or D2 allele remained constant across all cell types.

In total, we identified 365 probes that displayed a *static* eQTL at threshold *p*<10^−6^ (FDR = 0.02). Among the 268 *locally*-regulated probes in this category was *H2-D1*. The histocompatibility gene *H2-D1* is known to be polymorphic between B6 and D2 mice, and would therefore be expected to be in the *static* eQTL category. The remaining 97 probes mapped to *distant* eQTLs, i.e., their heritable expression variation was affected by the same *distant* locus in all four cell types (Table 1).

**Table 1:** Overview of *static* and *dynamic* eQTLs (*p*<10^−6^): number of probes and associated markers.

eQTL category	eQTL subcategory		# probes	# markers	# probes/# markers
***Static***	All	*Local*	268	161	1.66
		*Distant*	97	76	1.28
		Total	365	213	1.71
***Dynamic***	All	*Local*	642	282	2.28
		*Distant*	641	276	2.32
		Total	1283	445	2.88
	Stem-specific	*Local*	87	66	1.32
		*Distant*	53	42	1.26
		Total	140	105	1.33
	Progenitor-specific	*Local*	32	27	1.19
		*Distant*	13	12	1.08
		Total	45	39	1.15
	Erythroid-specific	*Local*	131	90	1.46
		*Distant*	400	164	2.44
		Total	531	223	2.38
	Myeloid-specific	*Local*	163	121	1.35
		*Distant*	132	72	1.83
		Total	295	179	1.65

All probes that belonged to the *static* eQTL category are graphically depicted in an eQTL dot plot displaying the genomic positions of the eQTLs compared to the genomic positions of the genes by which the variably expressed transcripts were encoded (Figure 2A, right panel). Whereas in this plot *local* eQTLs appear on the diagonal, *distant* eQTLs appear elsewhere. In general, as has been reported before in eQTL studies, transcripts that were *locally* regulated showed strong linkage statistics. Not surprisingly, the statistical association between genotype and variation in transcript abundance for those transcripts that were controlled by *distant* loci was weaker. These genes are likely to be controlled by multiple loci, each contributing only partially to the phenotype, thereby limiting their detection and validation in the current experimental sample size. A list of all transcripts with significant *static* eQTLs is provided in Table S2.

### Cell-Type–Dependent *Dynamic* eQTLs

The second eQTL category comprises genes that have *dynamic* eQTLs across all four cell types under study. In total, we identified 1283 eQTLs (*p*<10^−6^, FDR = 0.021) that showed different genetic effects in different cell types, indicating that eQTLs are highly sensitive to cellular differentiation state (Table 1). Within this *dynamic* eQTL category, the first four subcategories are composed of eQTLs that were preferentially active in only one of the four cell types we analyzed (Figures 2B–2E).

For example, *Slit2* mapped to a strong eQTL that was active only in stem cells. *Slit2* mRNA was only detected in the most primitive hematopoietic cell compartment in those BXD strains that carried the D2 allele at rs13478235, a SNP that mapped 629 kb away from the *Slit2* gene (Figure 2B, left panel). *Slit2* encodes an excreted chemorepellent molecule that is known to be expressed in embryonic stem cells [17], to be involved in neurogenesis [18] and angiogenesis [19], and to inhibit leukocyte chemotaxis [20]. We found a total of 140 genes that have eQTLs that are preferentially/selectively active in stem cells (Figure 2B, right panel, largest symbols, Table 1). These 140 genes included well-known candidate stem cell genes such as *Angpt1*, *Ephb2*, *Ephb4*, *Foxa3*, *Fzd6*, and *Hoxb5*. Interestingly, many transcripts with as yet unknown (stem cell) function were transcriptionally affected by stem-cell-specific eQTLs. Candidate novel stem cell genes include *Msh5*, and *Trim47*, in addition to a large collection of completely unannotated transcripts.

A total of 45, 531, and 295 eQTLs were found to be preferentially/selectively active in progenitors, erythroid cells, and myeloid cells, respectively (Table 1). Very distinct patterns of cell-type–specific gene regulation emerged when these eQTLs were visualized in genome-wide dot plots (Figures 2C–2E). Using genome-wide *p*-value thresholds of *p*<10^−6^, we identified 53 *distantly*-regulated transcripts in stem cells, 13 in progenitor cells, 400 in erythroid cells, and 132 in myeloid cells. In erythroid and myeloid cells most of these transcripts mapped to relatively few genomic loci; these trans-bands are statistically significant, as assessed by a permutation approach taking expression correlation into account (see Materials and Methods) [21]. Typically, transcripts mapping to a common marker showed a directional bias towards either B6 or D2 expression patterns.

In addition to the relatively simple eQTL dynamics that we have thus far illustrated, more complex eQTL dynamics were also detected using this approach. For example, *Rpo1-2* is a transcript that shows a strong *local* eQTL in the two non-committed lineages included in our study, but shows a much weaker genetic effect in erythroid and myeloid cells (Figure 2F). Whereas in mice carrying the B6 allele of *Rpo1-2* the overall expression of the gene decreased substantially during differentiation of progenitor to erythroid cells, in mice carrying the D2 allele expression slightly increased. This observation hints at complex regulatory mechanisms underlying the expression of this gene. Full lists of genes in each *dynamic* eQTL subcategory described thus far are supplied in Table S2. Additional subcategories and their exact definitions are explained more extensively in the Materials and Methods section, and complete results of all *dynamic* eQTLs are available in Table S3.

### Detailed Analysis of *Static* and *Dynamic* eQTLs

**Figure 3:**
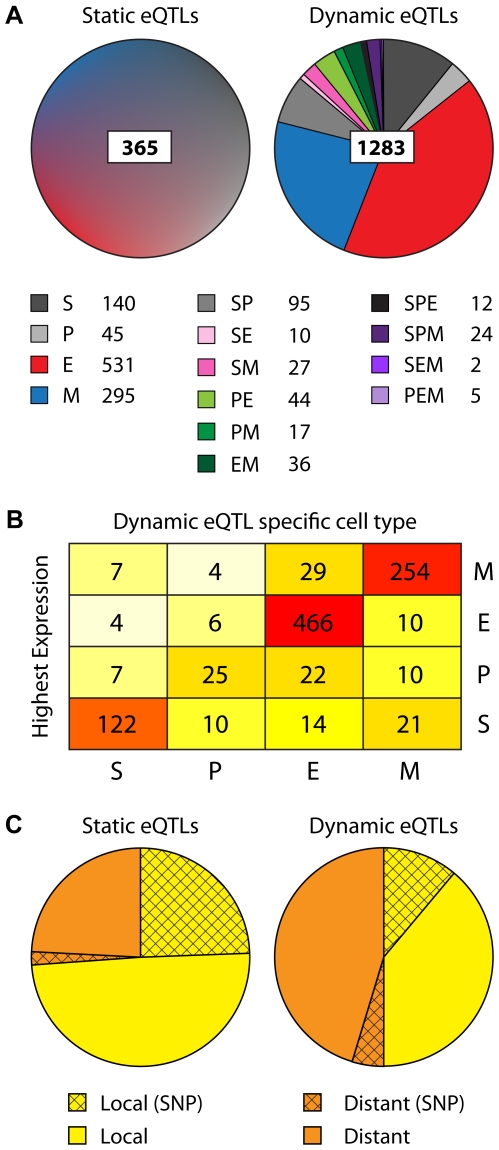
Quantitative overview of *static* and *dynamic* eQTLs. (A) Pie charts presenting all 365 *static* and 1283 *dynamic* eQTLs that were detected with *p*<10^−6^. *Dynamic* eQTLs are subdivided in all 14 categories of interaction eQTLs. (B) Matrix showing the four cell-type–dependent *dynamic* eQTL categories and the cell type in which the gene was expressed most highly. (C) All *static* and *dynamic* eQTLs are subdivided in *local* and *distant* eQTLs. Shown is which number of eQTLs was detected by Illumina probes that hybridize to sequences that are known to contain polymorphisms (SNPs) between the two parental strains. Abbreviations: S, stem cells; P, progenitor cells; E, erythroid cells; M, myeloid cells.

eQTL dynamics can be caused by transcription factors being switched on/off upon cellular differentiation, or by a transcription factor showing changed specificity due to variations in regulatory input. We found that most (>75%) of the *dynamic* eQTLs are active in only one of the four cell types under study (Figure 3A). A more detailed analysis revealed that in the majority of cases the genes with a cell-type–specific eQTL were also most highly expressed in that particular cell type (Figure 3B). Next, we explored whether we could find transcripts that were regulated by distinct eQTLs in different cell types (see Materials and Methods). Such eQTL “swapping” would indicate major changes in transcriptional regulatory networks. We could find no evidence for such cases. However, given our limited population size we have a low power to detect multiple eQTLs, so swapping eQTLs may still exist but remain undetected in our experimental setting.

It has been described that not all *local* eQTLs in genetical genomics experiments reflect actual expression differences between mouse strains, but rather indicate differential hybridization caused by polymorphisms in the sequences recognized by the probes [22]. For this reason, we divided both the *static* and *dynamic* eQTL categories in *local* and *distant* eQTLs, and indicated the number of probes that hybridized to sequences that are known to contain polymorphisms (Figure 3C). As expected, the *static* eQTL category contained a higher number of such potential false *local* eQTLs. If these false positive eQTLs could be removed, the relative abundance of *dynamic* eQTLs would be higher, indicating that our study may even conservatively underestimate the level of eQTL dynamics.

## Discussion

We found that many eQTLs are highly sensitive to the developmental state of the cell population under study. Even when the purified cells were only separated by a few cell divisions, eQTLs demonstrated a remarkable plasticity. Furthermore, we provide evidence that the cell-stage-sensitivity of eQTLs is often intertwined with gene expression variation during development. We did not identify target genes that were regulated by distinct eQTLs in different cell types, suggesting that large-scale changes within transcriptional regulatory networks are not common.

The fact that eQTLs appear to be highly cell-type–dependent highlights the importance of using well-characterized purified cell types in eQTL studies. In particular, eQTL studies of physiological or disease processes [23]–[26] should target the relevant cell type as precisely as possible, i.e. they should use cells or tissues directly involved in the patho-physiological process. This could even mean that several different cell types need to be separately studied, in particular if developmental trajectories are affected [27]. Using unfractionated bone marrow cells, we would have missed many of the diverse and dynamic patterns that we uncovered here, both at the expression level and at the genetic regulatory level. Even so, the four cell populations that we studied are still heterogeneous and further subfractionation of these populations based on different sets of markers would have resulted in even more precise regulatory maps.

Many genetical genomics experiments have used highly heterogeneous samples, in which mRNA from a variety of different cell types was pooled [4], [5], [28]–[31]. In such mixed samples it is usually impossible to ensure that the contribution of individual cell types to the mixture is the same across samples. As a result, important parts of the variation in gene expression could arise from different sample compositions. For example, if in whole brain samples a heritable morphological or developmental trait leads to an increased size of some brain regions, this can cause apparent hotspots for transcripts that are specific for those particular regions. Our data provide a valuable tool for studying the exact consequences of sample heterogeneity on eQTL mapping: a further study could simulate a collection of samples made of computed mixtures of different hematopoietic cells in defined proportions. Clearly, cell purification strategies are essential to identify those cell-type–specific eQTLs that would otherwise be “masked” in heterogeneous cell populations. Therefore, future genetical genomics studies should be realized on as many cell types or cellular differentiation states as possible, and ideally even on the scale of individual cells.

All data presented in this paper were deposited in the online database *GeneNetwork* (http://www.genenetwork.org), an open web resource that contains genotypic, gene expression, and phenotypic data from several genetic reference populations of multiple species (e.g. mouse, rat and human) and various cell types and tissues [32],[33]. It provides a valuable tool to integrate gene networks and phenotypic traits, and also allows cross-cell type and cross-species comparative gene expression and eQTL analyses. Our data can aid in the identification of candidate modulators of gene expression and/or phenotypic traits [34], and as such can serve as a starting point for hypothesis-driven research in the fields of stem cell biology and hematology.

## Materials and Methods

### Ethics Statement

All animal experiments were approved by the Groningen University Animal Care Committee.

### Recombinant Inbred Mice

Female BXD recombinant inbred mice were originally purchased from The Jackson Laboratory and housed under clean conventional conditions. Mice were used between 3 and 4 months of age.

### Cell Purification

Bone marrow cells were flushed from the femurs and tibias of three mice and pooled. After standard erythrocyte lysis, nucleated cells were stained with either a panel of biotin-conjugated lineage-specific antibodies (containing antibodies to CD3e, CD11b (Mac1), CD45R/B220, Gr-1 (Ly-6G and Ly-6C) and TER-119 (Ly-76)), fluorescein isothiocyanate (FITC)-conjugated antibody to Sca-1 and allophycocyanin (APC)-conjugated antibody to c-Kit, or with biotin-conjugated TER-119 antibody and FITC-conjugated antibody to Gr-1. After being washed, cells were incubated with streptavidin-phycoerythrin (PE) (all antibodies were purchased from Pharmingen). Cells were purified using a MoFlo flowcytometer (BeckmanCoulter) and were immediately collected in RNA lysis buffer. Lineage-depleted (Lin^−^) bone marrow cells were defined as the 5% of cells showing the least PE intensity.

### RNA Isolation and Illumina Microarrays

Total RNA was isolated using the RNeasy Mini kit (Qiagen) in accordance with the manufacturer's protocol. RNA concentration was measured using a Nanodrop ND-1000 spectrophotometer (Nanodrop Technologies). The RNA quality and integrity was determined using Lab-on-Chip analysis on an Agilent 2100 Bioanalyzer (Agilent Technologies). Biotinylated cRNA was prepared using the Illumina TotalPrep RNA Amplification Kit (Ambion) according to the manufacturer's specifications starting with 100 ng total RNA. Per sample, 1.5 µg of cRNA was used to hybridize to Sentrix Mouse-6 BeadChips (Illumina). Hybridization and washing were performed by ServiceXS according to the Illumina standard assay procedures. Scanning was carried out on the Illumina BeadStation 500. Image analysis and extraction of raw expression data were performed with Illumina Beadstudio v2.3 Gene Expression software with default settings and no normalization. The raw expression data from all four cell types were first log2 transformed and then quantile normalized as a single group.

### Clustering of Genes

For cluster analysis we retained only genes having a minimal fold change of 2 (difference of 1 in log2 scale) in either direction in mean expression on the transition from Lin^−^Sca-1^+^c-Kit^+^ to Lin^−^Sca-1^−^c-Kit^+^ and on the transition from Lin^−^Sca-1^−^c-Kit^+^ to TER-119^+^ or to Gr-1^+^. This filter reduced the dataset to 876 probes. We then computed the distance matrix for this group of probes, using the absolute Pearson correlation. Using this distance matrix, we applied the hierarchical clustering algorithm. From the resulting tree, 8 different clusters emerged from a manually chosen threshold. We then submitted each of these clusters to DAVID to identify enriched functional annotations [35].

### Full ANOVA Model for eQTL Mapping

The expression data of the four cell types were firstly corrected for batch effect and then analyzed separately by the following ANOVA model:
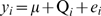
where *y_i_* is the gene's log intensity on the *i*th microarray; μ is the mean; Q*_i_* is the genotype effect under study; and *e_i_* is the residual error.

Next, expression data of the four cell types were combined and analyzed by a full ANOVA model including the cell type effect (CT) and the eQTL×CT interaction effect:
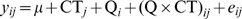
where *y_ij_* is the gene's log intensity at the *i*th microarray (*i* = 1,…*n*) and *j*th cell type; CT*_j_* is the *j*th cell type effect; (Q×CT)*_ij_* is the interaction effect between the *i*th eQTL genotype and *j*th cell type, and *e_ij_* is the residual error. The batch effect was included as one of the factors. For each probe, we performed a genome-wide linkage analysis to identify the two markers that showed the most significant main QTL effect and interaction effect, respectively.

### *Local* and *Distant* eQTLs

We defined an eQTL as *local* if it was located within less than 10 Mb from the gene. All other eQTLs were considered *distant*.

### Classification of eQTLs

The ANOVA yields significance *p*-values for the main QTL effect Q*_i_* and the interaction effect (Q×CT)*_ij_* for each probe at each marker. A small *p*-value for the interaction effect indicates that the eQTL effect is different between the cell types. This significant difference can be due to very diverse patterns, with different biological interpretations. It is therefore necessary to classify interaction eQTLs based on these patterns. To achieve this classification, for every interaction eQTL we evaluated the strength of the effect in each cell type by calculating the difference between the mean expression of both genotypes. The cell type for which the effect was the strongest was labeled “High.” The cell type whose effect was most different from the strongest effect was labeled “Low.” The remaining two cell types were assigned to the group they resembled most closely. This classification allowed us to define 14 categories of interaction eQTLs. Additionally, we identified eQTLs that have a consistent effect across all four cell types. This category of consistent eQTLs consists of all probes satisfying the following three conditions: the gene has a significant main effect Q*_i_* at marker *m*; for the same marker *m*, the interaction (Q×CT)*_ij_* is not significant; the mean eQTL effect across cell types has a coefficient of variation smaller than 0.3.

### Estimating the FDR for the Main QTL Effect

We permuted the strain labels in the genotype data 100 times, maintaining the correlation of expression traits while destroying any genetic association. Then we applied the full ANOVA model and stored the genome-wide minimum *p*-value for each transcript. Based on the resulting empirical distribution of *p*-values, we estimated that a threshold of −log_10_ *p* = 6 corresponds to a false discovery rate [36] of 0.02 for the main QTL effect. The 99.9th percentile of the number of significant eQTLs per marker (i.e., the minimum size of statistically significant “eQTL hotspots”) is 28.

### Estimating the FDR for Interaction QTL Effect

We estimated the residuals of the full ANOVA model after fitting all factors up to the main QTL effect at each marker for each transcript [37]. Then we permuted the strain labels and applied the ANOVA model y*_ij_* = Q*_i_* + CT*_j_* + (Q×CT)*_ij_* + *e_ij_* to the permuted residuals at each marker for each transcript and stored the genome-wide minimum *p*-value. Based on 100 permutations and the resulting empirical distribution of *p*-values, we estimated that a threshold of −log_10_ *p* = 6 corresponds to a false discovery rate of 0.021 for interacting QTL effect. The 99.9th percentile of the number of significant eQTLs per marker (i.e., the minimum size of statistically significant “interaction hotspots”) is 8.

### Detection of Swapping eQTLs

Swapping eQTLs are those transcripts that show one eQTL in one cell type, but another eQTL in another cell type. From the full model mapping described above, we obtained 1283 transcripts with a significant interaction effect between genotype (first marker) and cell type. After taking into account the genetic and interaction effects of the first marker, we scanned the genome excluding the region of the first marker (window size = 30 cM) and tested if there was a significant interaction effect between genotype and cell type and whether this new interaction effect was classified in a different cell type category (see above Classification of eQTLs), which would indicate a swapping eQTL.

This means, for each transcript, a two-marker full model mapping was applied using the following model:
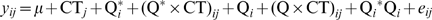
where *y_ij_* is the gene's log intensity at the *i*th microarray (*i* = 1,…*n*) and *j*th cell type; CT*_j_* is the *j*th cell type effect; Q^*^ *_i_* and (Q^*^×CT)*_ij_* are the main genotype effect at first marker and interaction effect between cell type and the genotype effect at this marker, where the first marker is defined as the marker with maximal interaction effect from previous one-marker full model mapping; Q*_i_* is the genotype effect of the second marker; (Q×CT)*_ij_* is the interaction effect between the *i*th genotype and *j*th cell type, Q*_i_* ^*^Q*_i_* is the epistasis effect and *e_ij_* is the residual error.

### URLs

All raw data were deposited in the NCBI Gene Expression Omnibus (GEO, http://www.ncbi.nlm.nih.gov/geo/, accession number GSE18067). All processed data were deposited in the GeneNetwork (http://www.genenetwork.org) [32],[33].

## Supporting Information

Figure S1 Analysis of the quantitative aspects of eQTLs. (A) Strain distribution pattern (expression values per strain) of *H2-Ob* transcript levels in stem cells. *H2-Ob* is a *locally* regulated transcript where only strains that carry the B6 allele of the gene (indicated by red bars) show gene expression. (B) As in panel A, but here *Apobec3* transcript levels are shown. (C) For all variably expressed transcripts genetic linkage analysis identified a genomic locus where presence of B6 or D2 alleles correlated with variation in expression levels of the corresponding gene. We compared the strength of the genetic association (eQTL effect) with the mean expression levels of the corresponding genes. Each dot refers to a single probe. If the eQTL effect is negative, B6 alleles at the locus most strongly associated with variation in transcript abundance increase its expression. If the eQTL effect is positive, D2 alleles at the eQTL increase expression. The data are shown for stem cells, but identical patterns were obtained for the other three cell populations. (D) This plot illustrates the size of the effect of the presence of either parental B6 or D2 allele at the eQTL on gene expression levels. Each dot refers to a single probe. For each probe expression values for strains carrying the B6 allele at the strongest associated marker were compared with values for strains carrying the D2 allele. Indicated in red are transcripts that are *locally* regulated by a strong eQTL mapping within 10 Mb from the corresponding gene.
(1.40 MB TIF)

Table S1 Clustering results.
(0.17 MB XLS)

Table S2 Principal eQTL categories.
(0.99 MB XLS)

Table S3 All *dynamic* eQTLs.
(0.87 MB XLS)
