# Supplementary figures and images for: Expression Quantitative Trait Loci Are Highly Sensitive to Cellular Differentiation State
Source: PLoS Genet. 2009 Oct 16;5(10):e1000692. doi: 10.1371/journal.pgen.1000692 (PMC2757904; doi:10.1371/journal.pgen.1000692)

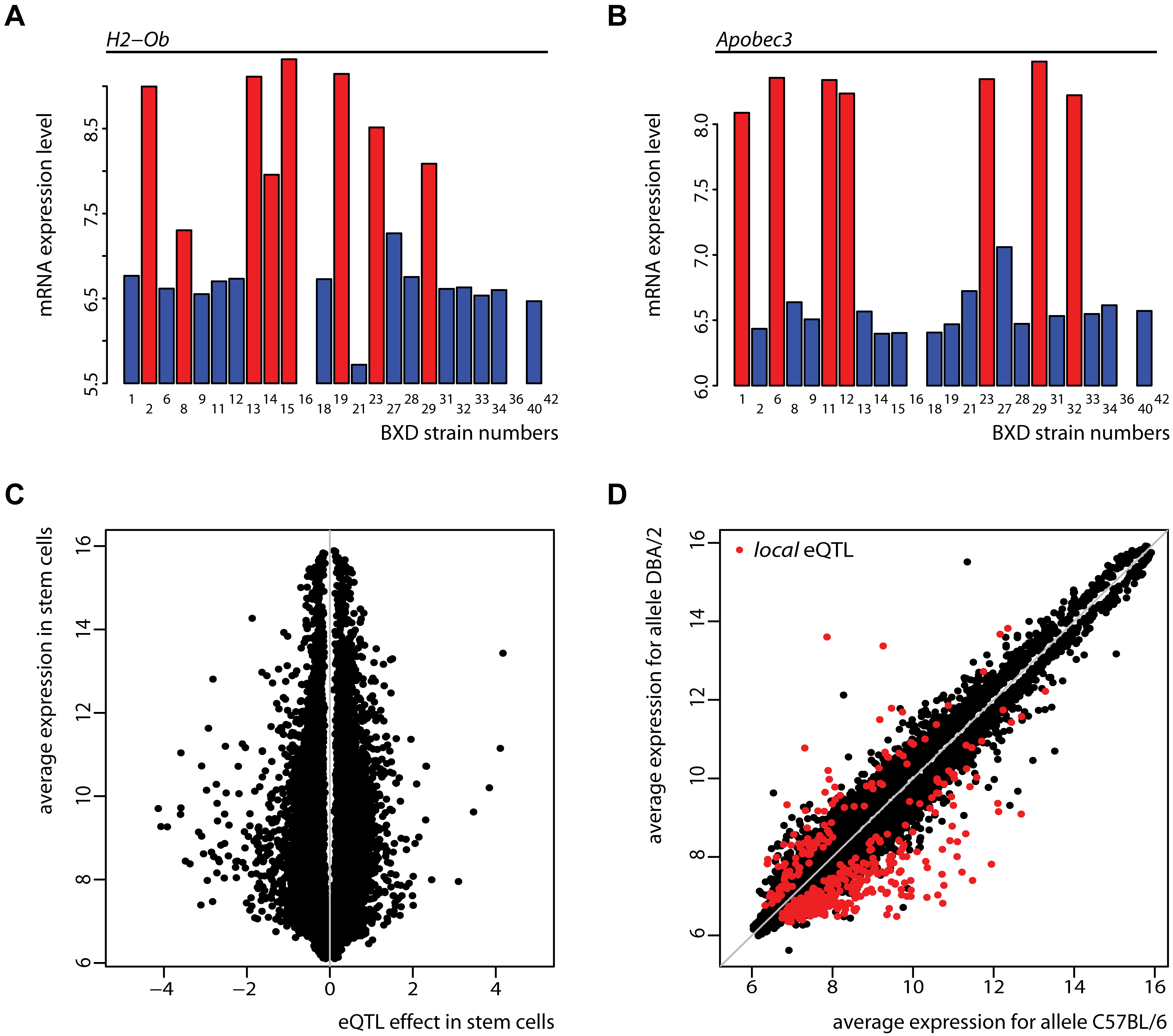

Supplement: Figure S1 — Analysis of the quantitative aspects of eQTLs. (A) Strain distribution pattern (expression values per strain) of H2-Ob transcript levels in stem cells. H2-Ob is a locally regulated transcript where only strains that carry the B6 allele of the gene (indicated by red bars) show gene expression. (B) As in panel A, but here Apobec3 transcript levels are shown. (C) For all variably expressed transcripts genetic linkage analysis identified a genomic locus where presence of B6 or D2 alleles correlated with variation in expression levels of the corresponding gene. We compared the strength of the genetic association (eQTL effect) with the mean expression levels of the corresponding genes. Each dot refers to a single probe. If the eQTL effect is negative, B6 alleles at the locus most strongly associated with variation in transcript abundance increase its expression. If the eQTL effect is positive, D2 alleles at the eQTL increase expression. The data are shown for stem cells, but identical patterns were obtained for the other three cell populations. (D) This plot illustrates the size of the effect of the presence of either parental B6 or D2 allele at the eQTL on gene expression levels. Each dot refers to a single probe. For each probe expression values for strains carrying the B6 allele at the strongest associated marker were compared with values for strains carrying the D2 allele. Indicated in red are transcripts that are locally regulated by a strong eQTL mapping within 10 Mb from the corresponding gene. (1.40 MB TIF) [file pgen.1000692.s001.tif]
